# Blood Stream Infections due to *Candida* Species: Prevalence, Risk Factors, Mortality, and Susceptibility Profile Over 3‐Year Period: A Single Center Study

**DOI:** 10.1155/ijm/6422952

**Published:** 2026-08-19

**Authors:** Ahmed Fahad Alduwayrij, Abdulmajeed AlArfaj, Maryam Alarbash, Mohammed Parvez, Omar Abdulmohsen Albanyan, Ibrahim Ahmed Alquhays, Ali AlMukhtar M. Alshanqiti, Yasser Abdulhalem Albaarrak, Sahar Eldirdiri, Muhammad Absar

**Affiliations:** ^1^ Department of Internal Medicine, King Abdulaziz Hospital, Ministry of the National Guard—Health Affairs, Al Ahsa, Saudi Arabia, ngha.med.sa; ^2^ Infection Prevention and Control Program, King Abdulaziz Hospital, Ministry of the National Guard—Health Affairs, Al Ahsa, Saudi Arabia, ngha.med.sa; ^3^ Intensive Care Unit, King Abdulaziz Hospital, Ministry of the National Guard—Health Affairs, Al Ahsa, Saudi Arabia, ngha.med.sa; ^4^ Prince Sultan Cardiac Center, Al Ahsa, Saudi Arabia, pscc.med.sa; ^5^ College of Medicine, Qassim University, Buraydah, Saudi Arabia, qu.edu.sa; ^6^ Department of Pathology and Laboratory Medicine, King Abdulaziz Hospital, Ministry of the National Guard—Health Affairs, Al Ahsa, Saudi Arabia, ngha.med.sa

**Keywords:** antifungals, *Candida* non-albicans, candidemia, risk factors, voriconazole

## Abstract

**Background:**

*Candida* is one of the most common causes of bloodstream infections in critically ill and immunocompromised patients. Understanding the distribution and antifungal resistance patterns of *Candida* species and the associated mortality rates is critical for tailoring therapeutic measures.

**Objectives:**

The aim of this study is to analyze the epidemiology, antifungal resistance trends, and risk factors influencing mortality associated with candidemia in a tertiary healthcare center of Saudi Arabia.

**Patient and Methods:**

The study included all cases of bloodstream infections caused by *Candida* species from December 2021 to January 2023. The antifungal susceptibility and identification of species were done via standard protocols. Univariate and multivariate statistical analyses were conducted to determine the predictors of 14‐day mortality (death within 14 days of the first positive blood culture) and overall (in‐hospital) mortality (death during the index hospitalization).

**Results:**

Fifty patients were included in the study with a median age of 71 years, with 58% being females. *C. albicans* accounted for 21.3% of candidemia cases, with a declining trend over time. *Candidozyma auris* (formerly *Candida auris*) was the most frequently identified non‐albicans *Candida* species (25%). Fluctuating trends in antifungal susceptibility of non‐albicans *Candida* were observed, with increasing resistance to azoles, while increasing susceptibility to micafungin. The 14‐day mortality and overall (in‐hospital) mortality rates were 23.4% and 55.3%, respectively. Multivariate analysis revealed older age, cardiac and respiratory disorders, ICU as site of diagnosis, treatment duration of > 48 h, and *Candida* colonization to be independent predictors of overall (in‐hospital) mortality (*p* < 0.05).

**Conclusion:**

The continuous rise of antifungal resistance and spread of non‐albicans *Candida* species highlight the need for implementing rigorous control measures in healthcare facilities. Improving candidemia outcomes requires prompt identification of high‐risk patients and targeted therapies.

## 1. Introduction

More than 90% of healthcare‐associated fungal bloodstream infections (BSI) are caused by *Candida* species (commonly referred to as candidemia) [1, 2]. Among BSIs reported in the United States and Europe, candidemia has been placed fourth and seventh, respectively, and third among neonatal late‐onset sepsis etiologies [3]. As opportunistic pathogens, *Candida* species cause severe systemic infections particularly in hospitalized and immunocompromised patients [4]. The global prevalence of *Candida* BSI is reported to be 0.3–5 per 1000 hospital admissions [5]. The rising incidence of candidemia has become a formidable concern in healthcare facilities, particularly in patients undergoing invasive medical treatments such as mechanical ventilation or central venous catheterization [6].

The epidemiology of candidemia differs among geographical regions according to differences in healthcare practices, antimicrobial usage, and patient population [7]. Although the most clinically predominant species associated with BSI has conventionally been *Candida albicans* (*C. albicans*), recently a progressive shift towards non‐albicans *Candida* species has been observed [2, 5]. These species, such as *C. glabrata*, *C. auris*, *C. tropicalis*, and *C. krusei*, usually show reduced susceptibility to common antifungals (azoles and echinocandins), challenging treatment strategies [8]. Recent taxonomic revisions have proposed reclassification of certain *Candida* species into new genera, including *Candidozyma auris* (formerly *C. auris*), *Nakaseomyces glabratus* (*C. glabrata*), and *Pichia kudriavzevii* (*C. krusei*) [9, 10]. Additionally, rare *Candida* or yeast infections cause significant diagnostic and therapeutic issues because they are difficult to identify using standard diagnostic laboratory tests based on phenotypic criteria such as growth on chromogenic media [4, 5]. Broad‐spectrum antibiotics and immunosuppressive therapeutics have been linked to the increased incidence of candidemia [11]. Old age, chemotherapy, mucositis, abdominal surgery, total parenteral nutrition (TPN), malignancy, neutropenia, as well as pancreatitis are other major risk factors for candidemia [6, 8].

Despite advances in antifungal therapies, candidemia has significantly high mortality rates (35%–71%), which vary according to patient comorbidities, the time of initiation of the antifungal drugs, and the compliance with infection control protocols [1, 12]. Prompt identification of risk factors, epidemiology and antifungal susceptibility patterns are essential to guide targeted therapeutic interventions for improving health outcomes [3, 7].

This study is aimed at comprehensively analyzing candidemia over a 3‐year period (2021–2023) in a single‐center setting. It particularly aimed at determining the risk factors, mortality rates, distribution of *Candida* species, antifungal susceptibility trends, patient outcomes, and resistance patterns associated with candidemia. Moreover, the study is aimed at highlighting the local epidemiology of candidemia to improve clinical management and reduce the burden of its complications in hospitalized patients.

## 2. Methodology

### 2.1. Study Setting, Design, and Population

This retrospective observational analysis was conducted at King Abdulaziz Hospital, Ministry of National Guard Health Affairs, Al Ahsa, Saudi Arabia, a secondary care center, over 3‐year duration from January 2021 to December 2023. The hospital can accommodate up to 400 beds and serves a diverse patient population. The data were obtained from hospitalized patients with BSI caused by the *Candida* species. The study was approved by the ethical committee (Study Approval #: NRA25/009/5) at King Abdullah International Medical Research Center (KAIMRC), and patient confidentiality was ensured according to the ethical guidelines.

The study initially included all patients with laboratory‐confirmed BSI caused by *Candida* species or *Trichosporon asahii*. Candidemia was defined as isolation of *Candida* from at least one blood culture accompanied by corresponding clinical symptoms and signs, with only the initial episode considered for analysis [13, 14]. Of the total study population (*n* = 50), 47 patients were diagnosed with *Candida* species, and three patients were diagnosed with *T. asahii*. The clinical and demographic features of *T. asahii* cases were not included in statistical analysis that focused on risk factors and outcomes associated with candidemia, but they were included in descriptive statistics.

Relevant demographic and clinical data of patients included: age; gender; comorbidities; treatment duration; severity of illness in the first 24 h following candidemia onset; antifungal exposure; hospital outcomes (14‐day and overall [in‐hospital]); underlying diseases and risk factors such as use of broad‐spectrum antibiotics, hemodialysis, hyperalimentation, immunosuppressive status, presence of invasive devices (e.g., CNS devices), neutropenia, and *Candida* colonization. No eligible patients were excluded owing to missing data. Sensitivity analysis was performed to evaluate the impact of minor missing data (if encountered) on the results.

Data regarding species identification and antifungal susceptibility profiles were obtained from the hospital microbiology laboratory database. Antifungal susceptibility profiles of identified species were determined following the Clinical and Laboratory Standards Institute (CLSI) guidelines [15]. Vitek 2 (bioMerieux, Inc., Hazelwood, Missouri, United States), an automated identification and susceptibility testing instrument, was used for all *Candida* species identification (YST card) and susceptibility testing (AST‐Y08 card).

### 2.2. Measured Outcomes

The primary outcomes included distribution of *Candida* species and associated risk factors for candidemia, whereas secondary outcomes included patterns of antifungal susceptibility, patient clinical outcomes, and mortality rates. Fourteen‐day mortality was defined as death from any cause within 14 days of the first positive blood culture for *Candida* species. Overall mortality was defined as all‐cause in‐hospital mortality during the index hospitalization [16]. Statistical analysis was used to evaluate risk factors for candidemia as well as mortality.

### 2.3. Statistical Analysis

SPSS Statistics (v27.0) software (IBM Corp, Armonk, New York, United States) was utilized for conducting all the statistical analyses in this study. Demographic and clinical characteristics of the patients were summarized using descriptive statistics. Continuous variables were presented as median and interquartile range (IQR) for nonnormally distributed data and mean ± standard deviation (SD) for normally distributed data. Normally distributed quantitative variables were compared using the independent *t-*test whereas nonnormally distributed ones were compared using the Mann–Whitney *U* test. Categorical variables were presented as frequencies and percentages. The chi‐square test was used to compare categorical data of two groups. Multivariate logistic regression analysis (backward stepwise model) was conducted to identify independent risk factors for candidemia patient outcomes. A two‐sided *p* value < 0.05 was considered statistically significant.

## 3. Results

### 3.1. Demographic and Clinical Profile of Patients

The median age of 50 enrolled patients was 71 years (IQR: 59.5–78). More than half (70%, *n* = 35) of the patients were > 60 years old, and majority of the patients were females (58%, *n* = 29), Table 1. A significant proportion of candidemia cases (58%, *n* = 29) were identified in intensive care units (ICUs), highlighting that critically ill patients are at higher risk. *C. auris* was the predominant cause of candidemia across all years followed by *C. albicans*. A total of 47 patients were included for the analysis of clinical characteristics of *Candida* infections excluding cases with *T. asahii* (*n* = 3), Table 2. The most prevalent comorbidities in candidemia patients were diabetes (70.2%, *n* = 33), cardiac diseases (57.4%, *n* = 27), renal failure (53.2%, *n* = 25), and other renal disorders (49%, *n* = 23). Moreover, the difference between *C. albicans* and *C.* non‐albicans group in age groups (in years), respiratory diseases, *Candida* colonization, and overall (in‐hospital) mortality was statistically significant (*p* value < 0.05), as shown in Table 2.

**Table 1:** Demographic characteristics of study participants, 2021–2023.

Variables		Overall	2021	2022	2023
Number of patients		50	22	14	14
Gender	Male	21 (42)	13 (59.1)	4 (28.6)	4 (28.6)
	Female	29 (58)	9 (40.9)	10 (71.4)	10 (71.4)

Age (in years)	Median (IQR)	71 (59.5–78)	69 (60–79.5)	66.5 (23–74)	75 (66–78)
Age groups (in years)	≤ 18	6 (12)	2 (9.1)	3 (21.4)	1 (7.1)
	19–59	6 (12)	3 (13.6)	3 (21.4)	0
	60–79	26 (52)	12 (54.5)	6 (42.9)	10 (71.4)
	≥ 80	9 (18)	5 (22.7)	2 (14.3)	3 (21.4)

Visit type	Emergency patients	3 (6)	1 (4.5)	2 (14.3)	0
	In‐patient	47 (94)	21 (95.5)	12 (85.7)	14 (100)

Site of isolation	ICU	29 (58)	16 (72.7)	7 (50)	6 (42.9)
	Non‐ICU	21 (42)	6 (27.3)	7 (50)	8 (57.1)

Organisms	*C. albicans*	10 (20)	4 (18.2)	3 (21.4)	3 (21.4)
	*C. tropicalis*	7 (14)	4 (18.2)	2 (14.3)	1 (7.1)
	*C. parapsilosis*	6 (12)	3 (13.6)	1 (7.1)	2 (14.3)
	*C. dubliniensis*	3 (6)	2 (9.1)	0	1 (7.1)
	*N. glabratus*	9 (18)	3 (13.6)	4 (28.6)	2 (14.3)
	*C. auris*	12 (24)	4 (18.2)	3 (21.4)	5 (35.7)
	*T. asahii*	3 (6)	2 (9.1)	1 (7.1)	0

14‐days mortality	Alive	36 (72)	15 (68.2)	11 (78.6)	10 (71.4)
	Dead	14 (28)	7 (31.8)	3 (21.4)	4 (28.6)

Overall (in‐hospital) mortality	Alive	21 (42)	8 (36.4)	6 (42.9)	7 (50)
	Dead	29 (58)	14 (63.6)	8 (57.1)	7 (50)

*Note:* Data represented as *n* (%) of patients or median (interquartile range), unless otherwise indicated.

Abbreviation: ICU, intensive care unit.

**Table 2:** Characteristics of patients with candidemia caused by *C.* non‐albicans and *C. albicans.*

Variables		Total (*n* = 47)	*C.* non‐albicans (*n* = 37)	*C. albicans* (*n* = 10)	Odds ratio (95% CI)	*p*
Age (years)		71 (58–78)	71 (62.5–79.5)	57.5 (7–74.8)	—	0.05
Age groups (in years)	≤ 18	6 (12.8)	2 (5.4)	4 (40)	0.32 (0.14–0.76)	**0.023**
	19–59	6 (12.8)	5 (13.5)	1 (10)		
	60–79	26 (55.3)	21 (56.8)	5 (50)		
	≥ 80	9 (19.1)	9 (24.3)	0		

Gender	Male	19 (40.4)	16 (43.2)	3 (30)	0.56 (0.13–2.52)	0.721
	Female	28 (59.6)	21 (56.8)	7 (70)		

Comorbidities	Diabetes	33 (70.2)	28 (75.7)	5 (50)	0.32 (0.08–1.37)	0.136
	Malignancy	6 (12.8)	5 (13.5)	1 (10)	0.71 (0.07–6.89)	1
	Renal diseases	23 (49)	21 (56.8)	2 (20)	0.19 (0.04–1.02)	0.067
	Cardiac diseases	27 (57.4)	24 (64.9)	3 (30)	0.23 (0.05–1.05)	0.067
	Respiratory diseases	13 (27.7)	13 (35.1)	0	1.79 (1.63–8.23)	**0.04**
	Liver diseases	4 (8.5)	3 (8.1)	1 (10)	1.26 (0.12–13.6)	1

Risk factors	Neutropenia	6 (12.8)	5 (13.5)	1 (10)	0.71 (0.07–6.89)	1
	Prolonged ICU stay	25 (53.2)	20 (54.1)	5 (50)	0.85 (0.21–3.44)	1
	Severe illness	35 (74.5)	28 (75.7)	7 (70)	0.75 (0.16–3.52)	0.69
	Broad‐spectrum antibiotics	45 (95.7)	35 (94.6)	10 (100)	0.68 (0.03–15.21)	1
	Immunosuppressive drugs	12 (25.5)	10 (27)	2 (20)	0.68 (0.12–3.74)	1
	Renal failure	25 (53.2)	22 (59.5)	3 (30)	0.29 (0.07–1.31)	0.147
	CNS devices	3 (6.4)	2 (5.4)	1 (10)	1.94 (0.16–23.92)	0.515
	Hyperalimentation	3 (6.4)	2 (5.4)	1 (10)	1.94 (0.16–23.92)	0.515
	Transplantation	1 (2.1)	1 (2.7)	0	0.86 (0.03–22.79)	1
	*Candida* colonization	14 (29.8)	14 (37.8)	0	1.96 (1.13–3.24)	**0.022**
	Hemodialysis	11 (23.4)	10 (27)	1 (10)	0.3 (0.03–2.68)	0.41

ICU (site of isolation)		26 (55.3)	20 (54.1)	6 (60)	1.28 (0.31–5.28)	1
Device related infection		19 (40.4)	13 (35.1)	6 (60)	2.77 (0.66–11.62)	0.279
Treatment duration	≤ 48 h	17 (36.2)	14 (37.8)	3 (30)	0.7 (0.16–3.18)	0.725
	> 48 h	30 (63.8)	23 (62.2)	7 (70)	1.98 (0.44–8.88)	0.475

Azole therapy		18 (38.3)	13 (35.1)	5 (50)	1.85 (0.45–7.57)	0.472
Mortality	14‐days mortality	11 (23.4)	10 (27)	9 (90)	3.33 (0.37–29.78)	0.414
	In‐hospital (overall) mortality	26 (55.3)	21 (56.8)	5 (50)	1.96 (1.32–5.42)	**0.031**

*Note:* Data represented as *n* (%) of patients or median (interquartile range), unless otherwise indicated. *p* value < 0.05 indicates statistical significance. Bold values indicate statistically significant differences (*p* < 0.05).

Abbreviations: CI, confidence interval; CNS, central nervous system; ICU, intensive care unit; OR, odds ratio.

We also performed comparative analysis of the clinical characteristics and outcomes of patients with *C. albicans* BSI with those of *C*. non‐albicans species (*C. dubliniensis*, *C. parapsilosis*, *C. tropicalis*, *C. auris*, and *N. glabratus*) identified in this study. Compared with *C. albicans*, *C. auris* BSI were associated with older patients (*p* value: 0.03) and had lower proportion of ≤ 18 years (*p* value: 0.02). Meanwhile, *C. auris* infected patients had higher proportion of respiratory diseases (*p* value: 0.04) and prior *Candida* colonization (*p* value: 0.02). Similarly, patients with *N. glabratus* BSI also had higher frequency of respiratory diseases (*p* value: 0.03) but lower rates of device related infections (*p* value: 0.04), relative to *C. albicans* group. *C. dubliniensis* BSI were also significantly associated with increased colonization (*p* value: 0.03) than *C. albicans*. In contrast, patients with *C. parapsilosis* and *C. tropicalis* BSI exhibited no significant differences compared with those infected with *C. albicans*. Mortality outcomes, including 14‐day and overall (in‐hospital) mortality were comparable across all *Candida* species. The results of these comparisons are also presented in Table S1.

### 3.2. Distribution and Temporal Trends of *Candida* Species

**Figure 1:**
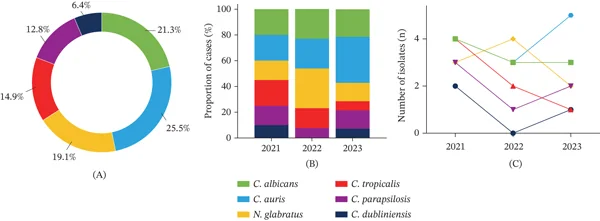
(A) Distribution (proportion, %) of the *Candida* species (*n* = 47) detected between 2021 and 2023. (B) Year‐wise proportion (%) of each *Candida* species. (C) Annual number of isolates (temporal trends) of each *Candida species* during the study period (2021–2023).

**Figure 2:**
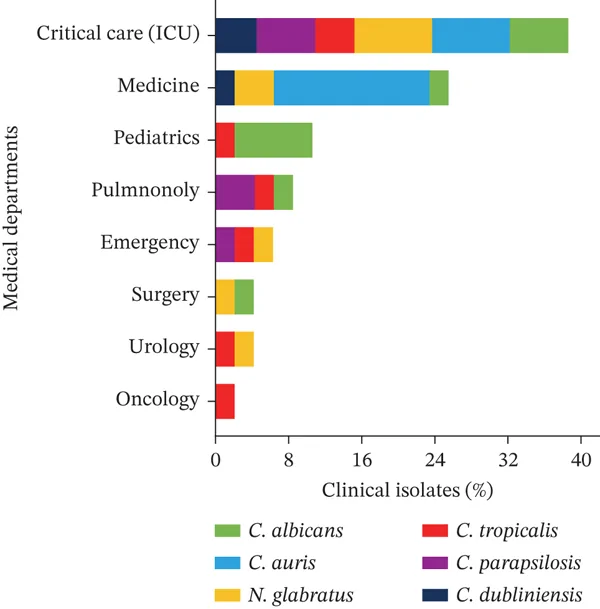
Total distribution of 47 *Candida* species across different hospital departments between January 2021 and December 2023.

During the 3‐year study period from 2021 to 2023, *C. albicans* accounted for 21.3% (*n* = 10) of the total 47 candidemia cases; however, non‐albicans *Candida* collectively represented 78.7% (*n* = 37). The most frequently identified non‐albicans species was *C. auris* (25.5%, *n* = 12), followed by *N. glabratus* (19.1%, *n* = 9) and *C. tropicalis* (14.9%, *n* = 7) (Figure 1A). Though *C. auris* dominated, the difference in the frequency between *C. albicans* and *C. auris* was not significant (*p* value: 0.76; 95% CI: −11.48 to 15.54). The temporal trends of *Candida* species also fluctuated over the study period, illustrated in Figure 1B,C. The proportion of *C. auris* increased over time, becoming the predominant species in 2023, whereas *C. tropicalis* showed a declining trend. Moreover, *C. dubliniensis* was identified sporadically throughout the study duration, whereas *C. albicans* remained relatively stable across all years (2021–2023). In contrast, *N. glabratus* and *C. parapsilosis* demonstrated fluctuating patterns without a consistent directional trend. Overall, the incidence of *Candida* BSIs varied over the 3‐year study period. During the first 2 years (2021–2022) of study, there was a decline in candidemia cases, increasing in the last year (2023) (Figure 2C).

Figure 2 illustrates the distribution of *Candida* species according to the patient ward. Overall, majority of the *Candida* species were from the critical care department (ICU) (36.2%, *n* = 17), followed by internal medicine department (25.5%, *n* = 12), pediatric department (10.6%, *n* = 5), pulmonology department (8.5%, *n* = 4), emergency department (6.4%, *n* = 3), urology department (4.3%, *n* = 2), surgery departments (both neurosurgery and vascular surgery departments: 4.3%, *n* = 2), and oncology department (2.1%, *n* = 1). Majority of *C. albicans* (40%, *n* = 4) were identified in the pediatric department, whereas the majority of *C. auris* (66.7%, *n* = 8) were identified in the internal medicine department.

**Figure 3:**
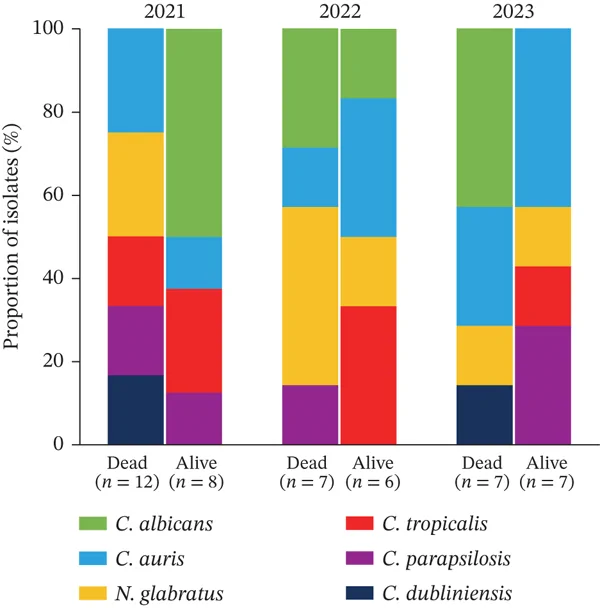
Temporal distribution of *Candida* species among deceased and surviving patients from 2021–2023.

Species distribution according to overall (in‐hospital) mortality revealed a predominance of non‐albicans *Candida* in both groups (alive and dead). Among patients who died during hospitalization (*n* = 26), non‐albicans *Candida* collectively accounted for 80.8% (*n* = 21), whereas *C. albicans* represented 19.2% (*n* = 5). Similarly, among surviving patients (*n* = 21), non‐albicans *Candida* comprised 76.2% (*n* = 16) of isolates compared with 23.8% (*n* = 5) for *C. albicans*. A significant difference was observed between *C. albicans* and non‐albicans *Candida* collectively (*p* value: 0.03; OR = 1.96, 95% CI: 1.32–5.42), Table 2. Temporal trends of *Candida* species distribution according to overall (in‐hospital) mortality stratified by year (2021–2023) are shown in Figure 3. Among deceased patients in 2021 (*n* = 12), *C. auris* and *N. glabratus* predominated (25% each, *n* = 3). *N. glabratus* also accounted for the largest proportion of isolates (42.9%, *n* = 3) in the dead group (*n* = 7) in 2022, whereas, in 2023 *C. albicans* became the most frequently identified species (42.9%, *n* = 3) among dead group (*n* = 7). Conversely, among surviving patients, *C. albicans* (50%, *n* = 4) predominated in 2021 (*n* = 8), whereas *C. auris* and *C. tropicalis* represented the largest proportion (33.3%, *n* = 2) in 2022 (*n* = 6). *C. auris* was also most prevalent (42.9%, *n* = 3) in survival group in 2023 (*n* = 7). However, no statistically significant differences were identified for individual species comparisons across years between both outcomes (surviving and dead).

### 3.3. Antifungal Susceptibility Pattern of Identified Species

Table 3 summarizes the sensitivity of *Candida* species to various antifungal agents determined through Vitek testing. Species‐specific differences in antifungal sensitivity were observed for fluconazole, amphotericin B, caspofungin, voriconazole, flucytosine, and anidulafungin, indicating that susceptibility patterns varied according to the *Candida* species (*p* value < 0.001). Although minimal variations in susceptibility to micafungin and itraconazole were observed, the difference was not statistically significant (*p* value > 0.05). Antifungal susceptibility testing revealed that 87.2% (*n* = 41) of the *Candida* species were susceptible to micafungin. *C.* albicans remained 100% susceptible to all the tested antifungals, except itraconazole and anidulafungin (Table 3). In the non‐albicans group, 83.8% (*n* = 31) strains were susceptible to micafungin, followed by amphotericin B (70.3%, *n* = 26), flucytosine (59.5%, *n* = 22), voriconazole (59.5%, *n* = 22), and flucytosine (35.1%, *n* = 13).

**Table 3:** Sensitivity of *Candida* species to various antifungal drugs.

Antifungal drugs	Overall (*n* = 47)	*C. albicans* (*n* = 10)	*C. auris* (*n* = 12)	*N. glabratus* (*n* = 9)	*C. tropicalis* (*n* = 7)	*C. parapsilosis* (*n* = 6)	*C. dubliniensis* (*n* = 3)	*p*
Fluconazole	23 (48.9)	10 (100)	0	0	7 (100)	5 (88.3)	1 (33.3)	**< 0.001**
Micafungin	41 (87.2)	10 (100)	8 (66.7)	9 (100)	6 (85.7)	6 (100)	2 (66.7)	0.068
Amphotericin B	36 (76.6)	10 (100)	3 (25)	9 (100)	7 (100)	5 (88.3)	1 (33.3)	**< 0.001**
Caspofungin	31 (66)	10 (100)	3 (25)	5 (55.6)	7 (100)	6 (100)	0	**< 0.001**
Voriconazole	32 (68.1)	10 (100)	0	9 (100)	7 (100)	4 (66.7)	2 (66.7)	**< 0.001**
Itraconazole	1 (2.1)	0	0	0	0	0	1 (33.3)	0.064
Flucytosine	32 (68.1)	10 (100)	0	8 (88.9)	7 (100)	6 (100)	1 (33.3)	**< 0.001**
Anidulafungin	10 (21.3)	0	8 (66.7)	0	0	0	2 (66.7)	**< 0.001**

*Note:* Data represented as *n* (%); *p* value < 0.05 indicates statistical significance. Bold values indicate statistically significant differences (*p* < 0.05).

A varying trend in susceptibility towards amphotericin B, caspofungin, and azoles (voriconazole and fluconazole) was observed among the *C.* non‐albicans over time during the study period (2021–2023), Figure 4. Fluconazole susceptibility declined progressively from 43.8% in 2021 to 30.0% in 2022 and further to 27.3% in 2023. A notable increase in susceptibility to micafungin was also observed among *C.* non‐albicans towards 2023. Caspofungin susceptibility also decreased over time, falling from 68.8% in 2021 to 36.4% in 2023. Similarly, flucytosine susceptibility declined from 68.8% to 45.5% during the study period (2021–2023).

**Figure 4:**
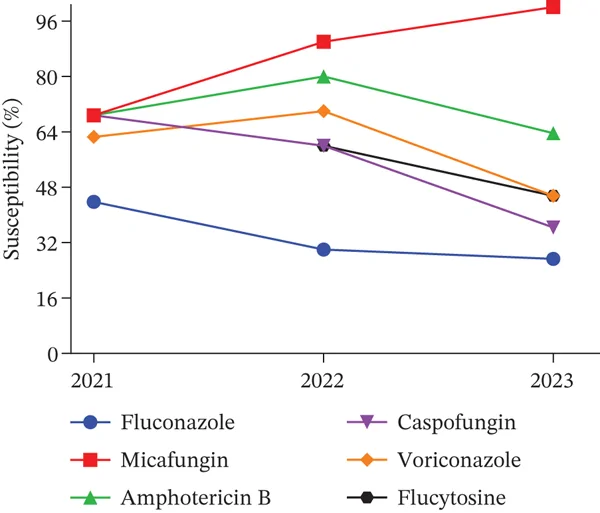
Antifungal susceptibility trend of *C.* non‐albicans species over time.

### 3.4. Factors Influencing Mortality and Patient Outcomes

**Figure 5:**
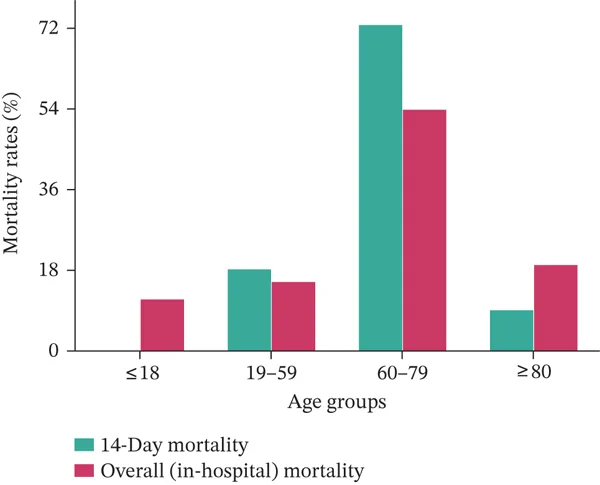
The 14‐day and overall (in‐hospital) mortality rates in candidemia patients of various age groups.

The overall (in‐hospital) mortality and 14‐day mortality were 55.3% (*n* = 26) and 23.4% (*n* = 11), respectively. The 14‐day mortality did not differ significantly by age groups (Figure 5). However, overall (in‐hospital) mortality varied by age groups; overall (in‐hospital) mortality was 11.5% (*n* = 3) in ≤ 18‐year age group, 15.4% (*n* = 4) in 19–59‐year age group, 53.8% (*n* = 14) in 60–79‐year age group, and 19.2% (*n* = 5) in ≥ 80‐year age group (Figure 5). This increase in mortality with increasing age category was significant (*p* value: 0.043).

**Figure 6:**
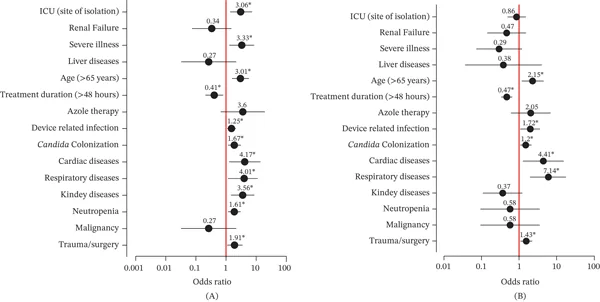
Unadjusted odds ratio and 95% CI of selected risk factors for (A) 14‐day mortality and (B) overall (in‐hospital) mortality among candidemia patients. The asterisk (*) indicates statistically significant differences (*p* < 0.05).

Univariate logistic regression analysis for predictors of 14‐day and overall (in‐hospital) mortality is represented in Figure 6A,B. Candidemia in patients with kidney diseases, cardiac diseases, and respiratory diseases resulted in increased 14‐day mortality (*p* < 0.05). Older age (> 65 years), severe illness, trauma/surgery, neutropenia, device‐related infections, *Candida* colonization, and ICU as the site of isolation were also significantly associated with higher 14‐day mortality (*p* < 0.05), Figure 6A. For overall (in‐hospital) mortality, candidemia in patients with trauma/surgery, respiratory diseases, and cardiac disorders was significantly associated with higher mortality (*p* < 0.05) (Figure 6B). Although prolonged ICU stay was observed in 53.2% (*n* = 25) of invasive candidemia cases, it was not significantly associated with 14‐day mortality or overall (in‐hospital) mortality. Additionally, device‐related infections, *Candida* colonization, and old age (> 65 years) also significantly increased odds of overall (in‐hospital) mortality in candidemia patients, Figure 6B. Prolonged treatment duration (> 48 h) was associated with significantly reduced odds of both 14‐day and overall (in‐hospital) mortality (Figure 6A,B). Results of univariate analysis for predictors of 14‐day and overall (in‐hospital) mortality are also summarized in Table S2.

Multifactorial logistic regression analysis also revealed independent predictors of 14‐day and overall (in‐hospital) mortality, shown in Table 4. After adjustment of potential confounders, cardiac diseases (*p* value: 0.02; adjusted odds ratio [AOR] = 5.54, 95% CI: 1.5–8.21), respiratory diseases (*p* value: 0.04; AOR = 3.67, 95% CI: 1.26–7.63), ICU as site of isolation (*p* value: 0.02; AOR = 2.35, 95% CI: 1.14–13.4), and *Candida* colonization (*p* value: 0.04; AOR = 1.81, 95% CI: 1.12–3.87) remained independently associated with increased odds of 14‐day mortality in candidemia patients (Table 4). For overall (in‐hospital) mortality, old age (> 65 years) (*p* value: 0.01; AOR = 2.13, 95% CI: 1.26–3.07) and *Candida* colonization (*p* value: 0.04; AOR = 2.13, 95% CI: 1.03–4.98) were significantly associated with two times increased odds of death (Table 4). Cardiac diseases (*p* value: 0.02; AOR = 2.63, 95% CI: 1.56–9.35) and respiratory diseases (*p* value: 0.01; OR = 2.43, 95% CI: 1.85–9.24) were also identified as independent predictors of overall (in‐hospital) mortality. However, candidemia patients receiving treatment for more than 48 h showed around 92% lower odds (*p* value: 0.02; AOR = 0.08, 95% CI: 0.01–0.7) of overall (in‐hospital) mortality as compared with those receiving treatment for less than 48 h (Table 4).

**Table 4:** Multivariate regression analysis of risk factors independently associated with 14‐day and overall (in‐hospital) mortality in patients with candidemia.

Risk factors	14‐days mortality			Overall (in‐hospital) mortality		
	AOR	95% CI	*p*	AOR	95% CI	*p*
Age (> 65 years)	1.05	0.98–1.12	0.139	2.13	1.26–3.07	**0.012**
Kidney diseases	4.38	0.36–6.33	0.247	0.92	0.14–5.99	0.925
Diabetes	2.47	0.31–11.04	0.334	0.81	0.1–6.25	0.836
Cardiac diseases	5.54	1.5–8.21	**0.021**	2.63	1.56–9.35	**0.015**
Respiratory diseases	3.67	1.26–7.63	**0.04**	2.43	1.85–9.24	**0.008**
ICU (site of isolation)	2.35	1.14–13.4	**0.023**	0.67	0.06–6.99	0.738
Severe illness	3.84	0.41–8.37	0.168	1.31	0.32–5.32	0.193
Treatment duration (> 48 h)	0.25	0.02–3.67	0.313	0.08	0.01–0.7	**0.018**
Device related infection	2.77	0.34–11.82	0.339	1.2	0.37–3.87	0.757
Neutropenia	0.28	0.03–2.64	0.265	0.49	0.14–1.73	0.269
Trauma/surgery	1.5	0.14–16.64	0.739	0.48	0.15–1.56	0.22
*Candida* colonization	1.81	1.12–3.87	**0.035**	2.13	1.03–4.98	**0.04**

*Note:* *p* value < 0.05 indicates statistical significance. Bold values indicate statistically significant differences (*p* < 0.05).

Abbreviations: AOR, adjusted odds ratio; CI, confidence interval; ICU, intensive care unit.

## 4. Discussion

We analyzed 47 candidemia cases at King Abdulaziz Hospital, Saudi Arabia, over a 3‐year period (January 2021 to December 2023). The study focused on the distribution and antifungal susceptibility patterns of *Candida* species, as well as clinical predictors of mortality in candidemia patients. Our findings are consistent with recent reports of shifting epidemiology as most patients had candidemia caused by non‐albicans *Candida* species (78.7%, *n* = 37) with *C. auris* being the most isolated single species (25.5%, *n* = 12). The increase in non‐albicans *Candida* species can be attributed to increased use of azole and other antifungal drugs over the last two decades, which exert selection pressure on *Candida* species and favor the growth of resistant organisms such as *C. auris* and *N. glabratus* [17]. Other geographical and ecological factors, characteristics of the infected patients, variations in monitoring and reporting protocols, and infection prevention and control strategies could also contribute to changing epidemiology of *Candida* species. Notably, *C. auris* emerged as a frequently isolated species in this study, whereas *C. tropicalis* has been the dominant species in previous reports [18, 19]. The high prevalence of *C. auris* observed in this cohort may be explained by its unique epidemiological traits that favor its persistence and spread within healthcare facilities, particularly ICUs [20]. Its ability to survive on environmental surfaces for longer periods along with decreased susceptibility to commonly used disinfectants facilitate the formation of persistent hospital reservoirs [21]. Additionally, *C. auris* demonstrates effective and prolonged colonization on human skin, which promotes continuous shedding and environmental contamination [22]. Unlike other *Candida* species, the predilection of *C. auris* for skin colonization increases its transmission via direct contact as well as through healthcare workers, and medical devices [23]. Concurrently, advanced laboratory diagnostic techniques such as MALDI‐TOF MS (matrix‐assisted laser desorption/ionization‐time of flight mass spectrometry) can now accurately identify *C. auris*, whereas earlier cases may have been misidentified as other *Candida* species [24]. The multidrug resistant nature of *C. auris* and its potential for healthcare‐associated outbreaks make its increasing prevalence in Saudi hospitals a formidable concern [25–27].

Comparative studies conducted in Saudi Arabia and neighboring Gulf countries demonstrate major variations in the epidemiology of candidemia, regarding the prevalence of non‐albicans *Candida*. In Saudi Arabia, geographical regions exhibit varying distributions of *Candida* species, likely due to population characteristics, comorbidities, and differing treatment strategies employed in each area, paralleling findings in America, Europe, and other Asian countries [28–30]. *C. albicans* has been the predominant cause of candidemia, particularly in women, in the central region of Saudi Arabia [11]. Contrarily, a shift has been observed in the increasing incidence of *C*. *auris* and *C. parapsilosis* cases in the western regions of Saudi Arabia in the past few years [25, 27]. Eastern regions report even greater diversity, with higher incidence rates of *N. glabratus*, *C. tropicalis*, and *C. auris*, consistent with our findings [18, 31]. *C. auris* has emerged as a significant pathogen causing life‐threatening BSI particularly in healthcare facilities in Qatar, Kuwait, and United Arab Emirates [32–34]. Although there is a considerable regional variation, the yearly incidence of candidemia may have stabilized or declined in some nations [12, 35]. However, we observed fluctuations in the number of candidemia cases (*n* = 47) in our study, decreasing from 20 in 2021 to 13 in 2022 and subsequently increasing to 14 in 2023. Increased use of echinocandins, institutional infection control practices, and variations in patient populations may have contributed to this shift over time [36].

In the current study, three isolates of *C. dubliniensis* were also identified representing 6.4% of the total *Candida* species. This finding is noteworthy given the rarity of *C. dubliniensis,* typically accounting for less than 2% of all diagnosed *Candida* BSI. A total of 33.3% (*n* = 1) of these patients had prior exposure to azole therapy, suggesting a potential role of selective pressure in its emergence. A review of clinical records of these patients also revealed underlying cardiovascular comorbidities (cardiac diseases, hypertension, and dyslipidemia), diabetes, severe illness, prolonged ICU stay, and a history of using broad‐spectrum antibiotics. Thus, impaired immunity (severe illness, diabetes), weakened vascular integrity (cardiac diseases), disrupted microbial barriers (broad‐spectrum antibiotics) along with prolonged exposure to nosocomial risk (ICU stay) in these patients may have created a favorable environment for *C. dubliniensis* to cause infection [37, 38]. These findings are consistent with previous studies reporting that despite its generally very low virulence, *C. dubliniensis* can cause superficial and systemic infections in immunocompromised patients with prior azole exposure [38, 39]. In the current study, statistical association could not be established due to small sample size (*n* = 3), but the observed descriptive pattern highlights the need for further investigation into the clinical and epidemiological determinants of BSIs caused by *C. dubliniensis*.

A major hazard to public health is the prevalence of antifungal resistance in *Candida* species [40]. This study shows a time‐related decline in azoles and echinocandins susceptibility in non‐albicans *Candida* species. The results are consistent with global surveillance data of increasing resistance rates, especially for *C. auris* and *C. tropicalis* [41]. Previous studies have also recorded increasing resistance rates among non‐albicans *Candida* species in Saudi Arabia, particularly against azoles and echinocandins [42, 43]. The increasing resistance of non‐albicans *Candida* species to fluconazole may be attributed to cumulative azole selective pressure and wide penetration of triazole drugs into tissues [44]. Due to its low adverse effects and reasonable price, fluconazole has become first choice of empirical or preventive drug for candidemia patients over the years [45]. Thus, its extensive clinical use favors the survival and proliferation of resistant *Candida* species. Similar declining trend in caspofungin susceptibility of non‐albicans *Candida* species possibly reflects the emergence of *FKS* gene mutations under echinocandins exposure [46]. Moreover, mutations in genes involved in cytosine permease and deaminase pathways decrease the susceptibility of non‐albicans *Candida* species to flucytosine; a trend also observed in our study [47]. The increasing rate of antifungal resistance demands the implementation of regular susceptibility testing to guide suitable therapeutic approaches [48]. A notable finding was the increasing susceptibility of non‐albicans *Candida* species to micafungin, from 68.8% in 2021 to 100% in 2023, which may reflect differential echinocandin usage or inherently greater micafungin susceptibility of species [43], although reduced susceptibility of *C. albicans* to fluconazole has been observed in several regions including Saudi Arabia [27, 49, 50]. However, it exhibited full susceptibility to all antifungals tested in the current study, highlighting a favorable treatment response for BSIs caused by this species. These results further underline the importance of regional epidemiological surveillance in effectively modifying and implementing antifungal stewardship protocols.

Several clinical and demographic factors were significantly associated with higher mortality in candidemia patients. Invasive candidemia is associated with high mortality, particularly in patients with comorbidities such as diabetes, renal impairment, respiratory, renal, and cardiac disorders [51–53]. Consistent with previous studies, severe illness, trauma/surgery, ICU as the site of isolation, respiratory diseases, kidney diseases, advanced age (> 65 years), neutropenia, device related infections, and *Candida* colonization were significant predictors of 14‐day mortality in this study [41, 54, 55]. Overall (in‐hospital) mortality was also significantly higher among patients who were old (> 65 years), and in those with device related infections. Candidemia patients with cardiac diseases, trauma/surgery, and respiratory diseases also had significantly higher overall (in‐hospital) mortality rates. *Candida* colonization was also significantly associated with worse overall (in‐hospital) outcome as it may have been the reason for *Candida* entering the bloodstream, leading to infection. Notably, treatment duration of > 48 hours was significantly associated with reduced risk of overall (in‐hospital) mortality highlighting that adequate duration of antifungal therapy is crucial for increasing patients′ survival. Hayashi et al. [56] also reported similar findings that administration of antifungal drugs, particularly echinocandins, within 72 h of the initial fever can significantly increase survival rates of candidemia patients.

In our study, cardiac diseases emerged as one of the critical independent predictors of mortality, with affected patients experiencing sixfold‐increased risk for 14‐day mortality and threefold‐increased risk for overall (in‐hospital) mortality even after adjustment of other significant variables (Table 4). Cardiovascular disorders substantially compromise hemodynamic stability, reduce immune cell trafficking and disrupt tissue perfusion, reducing patient chances of surviving the inflammatory burden of candidemia and fungal sepsis [57]. Previous studies have also identified cardiac diseases as a major independent risk factor for mortality in candidemia patients with odds ratio as high as 19.36 [58, 59]. In addition, 74.5% (*n* = 35) of our study comprised patients 60 years and older, and thereby, were particularly vulnerable to invasive candidemia and chronic organ failure due to immune senescence.

Binkhamis et al. [60] reported in their retrospective analysis of 236 ICU patients in a tertiary care center in Riyadh that prolonged hospitalization, comorbidities, advanced age, as well as COVID‐19 infections led to severe clinical outcomes and higher mortality rates in candidemia patients. In South Africa, COVID‐19 pandemic, particularly Delta wave, might have been major driver for candidemia cases caused by *C. auris* increasing from 17% in 2019 to 24% in 2020, 31% in 2021, and 28% in 2022 [61].

Despite a high overall (in‐hospital) mortality rate (55.3%, *n* = 26) in our cohort, no significant difference was observed between various *Candida* species. However, patients with *Candida* BSIs caused by non‐albicans *Candida* species (collectively) had around two times higher odds of deaths (overall mortality) as compared with those caused by *C. albicans* (*p* value: 0.03). Studies have reported that *C. tropicalis* infections are associated with the lowest survival rates whereas *C. auris* exhibits the highest survival rates [62]. Contrary to previous findings, azole therapy was not significantly associated with 14‐day or overall (in‐hospital) mortality in our study [16, 63, 64]. This discrepancy may be attributed to the limited sample size of this study. Notably, a lower proportion of death was observed among azole‐treated patients in both 14‐day mortality (18.2%, *n* = 2) and overall (in‐hospital) mortality (30.8%, *n* = 8) compared with those who did not receive azole therapy. This finding also highlights potentially protective trend that requires further validation in larger cohort settings. Antifungal selection alone cannot determine mortality outcome of candidemia patients independently of other clinical factors including comorbidities, disease severity, multiorgan dysfunction, and time‐to‐diagnosis [65]. Moreover, increasing azole resistance observed among non‐albicans *Candida* species over the study period from 2021 to 2023 may also have influenced treatment outcomes, as administration of azoles to patients infected with resistant *Candida* species carries a high risk of therapeutic failure [66]. In this context, current IDSA and ESCMID guidelines suggest use of echinocandins as the first‐line therapy for candidemia, especially when the patient is critically ill or infected with non‐albicans *Candida*, due to their broad‐spectrum fungicidal activity and good safety profile [67].

The limited generalizability of this study is attributable to its single‐center, retrospective cohort design. Additionally, the study did not consider the differences between patient management strategies across various healthcare facilities, which could influence patient outcomes. More comprehensive national data, encompassing multiple healthcare facilities and regions, is required.

This retrospective study, nevertheless, provides valuable insights into the resistance patterns, epidemiological distribution, and clinical outcomes of candidemia. In conclusion, non‐albicans *Candida* was the leading cause of invasive candidemia, exhibiting temporal variation with a rising trend in later years, whereas the proportion of *C. albicans* decreased. The decline in antifungal susceptibility and increasing burden of non‐albicans *Candida* in clinical settings demands rigorous surveillance and antimicrobial stewardship strategies.

## Author Contributions

**Muhammad Absar:** conceptualization, data curation, writing – original draft, writing – review and editing. **Ahmed Fahad Alduwairij:** conceptualization, data curation, writing – original draft, writing – review and editing. **Abdulmajeed AlArfaj:** conceptualization, writing – original draft, writing – review and editing. **Maryam Alarbash:** data curation. **Mohammed Parvez:** data curation, writing – original draft, writing – review and editing. **Omar Abdulmohsen Albanyan:** data curation. **Ibrahim Ahmed Alquhays:** data curation. **Ali AlMukhtar M. Alshanqiti:** data curation. **Yasser Abdulhalem Albaarrak:** data curation. **Sahar Eldirdiri:** data curation, writing – review and editing.

## Funding

No funding was received for this manuscript.

## Disclosure

All authors have read and agreed to the published version of the manuscript.

## Conflicts of Interest

The authors declare no conflicts of interest.

## Supporting information


**Supporting Information** Additional supporting information can be found online in the Supporting Information section. Table S1: Comparison of clinical characteristics among patients with bloodstream infection (BSI) caused by major *Candida* species. Table S2: Univariate analysis of factors associated with 14‐day and overall (in‐hospital) mortality in patients with candidemia.

## Data Availability

The data that support the findings of this study are available from the corresponding author upon reasonable request.
