# Supplementary material for: Blood Stream Infections due to Candida Species: Prevalence, Risk Factors, Mortality, and Susceptibility Profile Over 3‐Year Period: A Single Center Study
Source: Int J Microbiol. 2026 Aug 19;2026:6422952. doi: 10.1155/ijm/6422952 (PMC13490827; doi:10.1155/ijm/6422952)
Supplement: Supplementary file 1 [file IJM-2026-6422952-s001.docx]

**Supplementary File**

***Supplementary Table S1. Comparison of clinical characteristics among patients with BSI caused by major Candida species***

| **Variables** | | **Total (47)** | **CA**  **(10)** | **CD**  **(3)** | **CP**  **(6)** | **CT**  **(7)** | **CAu**  **(12)** | **NG**  **(9)** | **CA vs NG** | **CA vs CT** | **CA vs CP** | **CA vs CD** | **CA vs Cau** |
| --- | --- | --- | --- | --- | --- | --- | --- | --- | --- | --- | --- | --- | --- |
| Age | | 71 (58-78) | 57.5 (7-74.8) | 71 (R: 68-71) | 73 (65.5-89.5) | 58 (6-78) | 71 (64-80.8) | 72 (59-82) | 0.09 | 0.96 | 0.09 | 0.61 | **0.03** |
| Age groups (in years) | ≤18 | 6 (12.8) | 4 (40) | 0 | 0 | 2 (28.6) | 0 | 0 | 0.11 | 0.52 | 0.08 | 0.61 | **0.02** |
|  | 19–59 | 6 (12.8) | 1 (10) | 0 | 0 | 2 (28.6) | 1 (8.3) | 2 (22.2) |  |  |  |  |  |
|  | 60–79 | 26 (55.3) | 5 (50) | 3 (100) | 4 (66.7) | 2 (28.6) | 7 (58.3) | 5 (55.6) |  |  |  |  |  |
|  | ≥80 | 9 (19.1) | 0 | 0 | 2 (33.3) | 1 (14.3) | 4 (33.3) | 2 (22.2) |  |  |  |  |  |
| Gender | Male | 19 (40.4) | 3 (30) | 0 | 3 (50) | 3 (42.9) | 3 (25) | 7 (77.8) | 0.07 | 0.64 | 0.61 | 0.53 | 1 |
|  | Female | 28 (59.6) | 7 (70) | 3 (100) | 3 (50) | 4 (57.1) | 9 (75) | 2 (22.2) |  |  |  |  |  |
| Comorbidities | Diabetes | 33 (70.2) | 5 (50) | 3 (100) | 6 (100) | 3 (42.9) | 9 (75) | 7 (77.8) | 0.35 | 1 | 0.09 | 0.23 | 0.38 |
|  | Malignancy | 6 (12.8) | 1 (10) | 1 (33.3) | 1 (16.7) | 1 (14.3) | 1 (8.3) | 1 (11.1) | 0.47 | NA | 1 | 0.42 | 1 |
|  | Renal diseases | 23 (49) | 2 (20) | 2 (66.7) | 4 (66.7) | 3 (42.9) | 6 (50) | 6 (66.7) | 0.07 | 0.59 | 0.12 | 0.2 | 0.2 |
|  | Cardiac diseases | 27 (57.4) | 3 (30) | 3 (100) | 3 (50) | 3 (42.9) | 9 (75) | 6 (66.7) | 0.18 | 0.64 | 0.61 | 0.07 | 0.08` |
|  | Respiratory diseases | 13 (27.7) | 0 | 1 (33.3) | 2 (33.3) | 1 (14.3) | 5 (41.7) | 4 (44.4) | **0.03** | 0.41 | 0.13 | 0.23 | **0.04** |
|  | Liver diseases | 4 (8.5) | 1 (10) | 1 (33.3) | 0 | 2 (28.6) | 0 | 0 | 1 | 0.53 | 1 | 0.42 | 0.46 |
| Risk factors | Neutropenia | 6 (12.8) | 1 (10) | 0 | 0 | 1 (14.3) | 1 (8.3) | 3 (33.3) | 0.3 | 1 | 1 | 1 | 1 |
|  | Prolonged ICU stay | 25 (53.2) | 5 (50) | 2 (66.7) | 4 (66.7) | 2 (28.6) | 8 (66.7) | 4 (44.4) | 1 | 0.62 | 0.63 | 1 | 0.67 |
|  | Severe illness | 35 (74.5) | 7 (70) | 3 (100) | 4 (66.7) | 4 (57.1) | 10 (83.3) | 7 (77.8) | 1 | 0.64 | 1 | 0.53 | 0.63 |
|  | Broad-spectrum antibiotics | 45 (95.7) | 10 (100) | 3 (100) | 6 (100) | 6 (85.7) | 11 (91.7) | 9 (100) | - | 0.41 | NA | NA | 1 |
|  | Immunosuppressive drugs | 12 (25.5) | 2 (20) | 1 (33.3) | 1 (16.7) | 3 (42.9) | 3 (25) | 2 (22.2) | 1 | 0.59 | 1 | 1 | 1 |
|  | Renal Failure | 25 (53.2) | 3 (30) | 2 (66.7) | 4 (66.7) | 2 (28.6) | 8 (66.7) | 6 (66.7) | 0.18 | 1 | 0.3 | 0.51 | 0.2 |
|  | CNS devices | 3 (6.4) | 1 (10) | 0 | 0 | 1 (14.3) | 0 | 1 (11.1) | 1 | 1 | 1 | 1 | 0.46 |
|  | Hyperalimentation | 3 (6.4) | 1 (10) | 0 | 0 | 2 (28.6) | 0 | 0 | 1 | 0.54 | 1 | 1 | 0.46 |
|  | Transplantation | 1 (2.1) | 0 | 0 | 0 | 0 | 0 | 1 (11.1) | 0.47 | NA | NA | NA | NA |
|  | *Candida* colonization | 14 (29.8) | 0 | 2 (66.7) | 2 (33.3) | 2 (28.6) | 6 (50) | 2 (22.2) | 0.21 | 0.15 | 0.13 | **0.03** | **0.02** |
|  | Hemodialysis | 11 (23.4) | 1 (10) | 2 (66.7) | 2 (33.3) | 2 (28.6) | 3 (25) | 1 (11.1) | 1 | 0.54 | 0.52 | 0.11 | 0.6 |
| ICU (site of isolation) | | 26 (55.3) | 6 (60) | 2 (66.7) | 4 (66.7) | 2 (28.6) | 7 (58.3) | 5 (55.6) | 1 | 0.34 | 1 | 1 | 1 |
| Device related infection | | 19 (40.4) | 6 (60) | 3 (100) | 3 (50) | 3 (42.9) | 3 (25) | 1 (11.1) | **0.04** | 0.64 | 1 | 0.5 | 0.19 |
| Treatment duration | ≤ 48 hours | 17 (36.2) | 3  (30) | 2 (66.7) | 3  (50) | 1 (14.3) | 6  (50) | 2 (22.2) | 1 | 0.61 | 0.61 | 0.51 | 0.42 |
|  | > 48 hours | 30 (63.8) | 7 (70) | 1 (33.3) | 3 (50) | 6 (85.7) | 5 (41.7) | 5 (55.6) | 0.65 | 0.6 | 0.61 | 0.51 | 0.23 |
| Azole therapy | | 18 (38.3) | 5  (50) | 1 (33.3) | 3  (50) | 5 (71.4) | 3 (25) | 1 (11.1) | 0.14 | 0.62 | 1 | 1 | 0.38 |
| Mortality | 14-days mortality | 11 (23.4) | 1  (10) | 2 (66.7) | 0 | 2 (28.6) | 3 (25) | 3 (33.3) | 0.3 | 0.54 | 1 | 0.11 | 0.6 |
|  | In-hospital (overall) mortality | 26 (55.3) | 5  (50) | 3  (100) | 3  (50) | 2 (28.6) | 6 (50) | 7 (77.8) | 0.35 | 0.62 | 1 | 0.23 | 1 |

CA: *C. albicans*; CD: *C. dubliniensis*; CP: *C. parapsilosis*; CT: *C. tropicalis*; Cau: *C. auris*; NG: *N. glabratus*; p-value <0.05 indicates statistical significance.

***Supplementary Table S2*. Univariate analysis of factors associated with 14-day and overall (in-hospital) mortality in patients with candidemia.**

| **Variables** | | **14-day outcome** | | | **Overall (in-hospital) mortality** | | |
| --- | --- | --- | --- | --- | --- | --- | --- |
|  |  | **Alive (*n*= 36)** | **Dead (*n*= 11)** | **p-value** | **Alive (*n*= 21)** | **Dead (*n* = 26)** | **p-value** |
| Age | | 73 (51.3-78.8) | 68 (60-72) | 0.41 | 74 (57.5-78) | 68.5 (7-78.3) | 0.507 |
| Age groups (in years) | ≤18 | 6 (16.7) | 0 | 0.38 | 3 (14.3) | 3 (11.5) | **0.043** |
|  | 19–59 | 4 (11.1) | 2 (18.2) |  | 2 (9.5) | 4 (15.4) |  |
|  | 60–79 | 18 (50) | 8 (72.7) |  | 12 (57.1) | 14 (53.8) |  |
|  | ≥80 | 8 (22.2) | 1 (9.1) |  | 4 (19.1) | 5 (19.2) |  |
| Gender | Male | 14 (38.9) | 5 (45.5) | 0.74 | 12 (57.1) | 16 (61.5) | 0.775 |
|  | Female | 22 (61.1) | 6 (54.5) |  | 9 (42.9) | 10 (38.5) |  |
| Primary diagnosis | Abdominal pathology | 20 (55.6) | 3 (27.3) | 0.17 | 12 (57.1) | 11 (42.3) | 0.385 |
|  | Trauma/surgery | 5 (13.9) | 1 (9.1) | **0.04** | 4 (19) | 2 (7.7) | **0.04** |
|  | Sepsis/infection | 30 (83.3) | 11 (100) | 0.31 | 16 (76.2) | 25 (96.2) | 0.076 |
|  | Kidney diseases | 13 (36.1) | 9 (81.8) | **0.01** | 7 (33.3) | 15 (57.7) | 0.143 |
| Comorbidities | Diabetes | 22 (61.1) | 11 (100) | **0.02** | 13 (61.9) | 20 (76.9) | 0.342 |
|  | Malignancy | 2 (5.6) | 4 (36.4) | 0.23 | 0 | 4 (15.4) | 0.117 |
|  | Cardiac diseases | 17 (47.2) | 10 (90.9) | **0.01** | 8 (38.1) | 19 (73.1) | **0.021** |
|  | Respiratory diseases | 7 (19.4) | 6 (54.5) | **0.04** | 1 (4.8) | 12 (46.2) | **0.002** |
|  | Liver diseases | 2 (5.6) | 2 (18.2) | 0.23 | 1 (4.8) | 3 (11.5) | 0.617 |
| Risk factors | Neutropenia | 5 (13.9) | 1 (9.1) | **0.04** | 2 (9.5) | 4 (15.4) | 0.678 |
|  | Prolonged ICU stay | 18 (50) | 7 (63.6) | 0.51 | 10 (47.6) | 15 957.7) | 0.564 |
|  | Severe illness | 25 (69.4) | 10 (90.9) | **0.02** | 13 (61.9) | 22 (84.6) | 0.1 |
|  | Broad-spectrum antibiotics | 34 (94.4) | 11 (100) | 1 | 19 (90.5) | 26 (100) | 0.194 |
|  | Immunosuppressive drugs | 8 (22.2) | 4 (36.4) | 0.44 | 5 (23.8) | 7 (26.9) | 1 |
|  | Renal Failure | 17 (47.2) | 8 (72.7) | 0.18 | 9 (42.9) | 16 (61.5) | 0.248 |
|  | Hyperalimentation | 3 (8.3) | 0 | 1 | 3 (14.3) | 0 | 0.082 |
|  | *Candida* colonization | 10 (27.8) | 4 (36.4) | **0.01** | 6 (28.6) | 8 (30.8) | **<0.001** |
|  | Hemodialysis | 7 (19.4) | 4 (36.4) | 0.26 | 3 (14.3) | 8 (30.8) | 0.3 |
| ICU (site of isolation) | | 15 (41.7) | 11 (100) | **<0.001** | 9 (42.9) | 17 (65.4) | 0.149 |
| Device related infection | | 15 (41.7) | 4 (36.4) | **0.01** | 10 (47.6) | 9 (34.6) | **0.04** |
| Treatment duration | ≤ 48 hours | 14 (38.9) | 3 (27.3) | 0.72 | 11 | 6 (23.1) | 0.066 |
|  | > 48 hours | 21 (58.3) | 6 (54.5) | **0.01** | 10 (47.6) | 17 (65.4) | **0.02** |
| Azole therapy | | 16 (44.4) | 2 (18.2) | 0.16 | 10 (47.6) | 8 (30.8) | 0.366 |
| Organisms | *C. albicans* | 9 (25) | 1 (9.1) | 0.27 | 5 (23.8) | 5 (19.2) | 0.289 |
|  | *N. glabratus* | 6 (16.7) | 3 (27.3) |  | 2 (9.5) | 7 (26.9) |  |
|  | *C. tropicalis* | 5 (13.9) | 2 (18.2) |  | 5 (23.8) | 2 (7.7) |  |
|  | *C. parapsilosis* | 6 (16.7) | 0 |  | 3 (14.3) | 3 (11.5) |  |
|  | *C. dubliniensis* | 1 (2.8) | 2 (18.2) |  | 0 | 3 (11.5) |  |
|  | *C. auris* | 9 (25) | 3 (27.3) |  | 6 (28.6) | 6 (23.1) |  |

Abbreviations: ICU, intensive care unit; *p*-value <0.05 indicates statistical significance.
